# Fear-induced brain activations distinguish anxious and trauma-exposed brains

**DOI:** 10.1038/s41398-020-01193-7

**Published:** 2021-01-13

**Authors:** Zhenfu Wen, Marie-France Marin, Jennifer Urbano Blackford, Zhe Sage Chen, Mohammed R. Milad

**Affiliations:** 1 Department of Psychiatry, New York University School of Medicine, New York, NY USA; 2 Department of Psychology, Université du Québec à Montréal & Research Center of the Institut Universitaire en Santé Mentale de Montréal, Montreal, QC Canada; 3 Department of Psychiatry and Behavioral Sciences, Vanderbilt University Medical Center, Nashville, TN USA; 4 Tennessee Valley Healthcare Services, Department of Veterans Affairs, Nashville, TN USA; 5 Department of Neuroscience and Physiology, New York University School of Medicine, New York, NY USA; 6 The Neuroscience Institute, New York University School of Medicine, New York, NY USA

**Keywords:** Psychiatric disorders, Diagnostic markers

## Abstract

Translational models of fear conditioning and extinction have elucidated a core neural network involved in the learning, consolidation, and expression of conditioned fear and its extinction. Anxious or trauma-exposed brains are characterized by dysregulated neural activations within regions of this fear network. In this study, we examined how the functional MRI activations of 10 brain regions commonly activated during fear conditioning and extinction might distinguish anxious or trauma-exposed brains from controls. To achieve this, activations during four phases of a fear conditioning and extinction paradigm in 304 participants with or without a psychiatric diagnosis were studied. By training convolutional neural networks (CNNs) using task-specific brain activations, we reliably distinguished the anxious and trauma-exposed brains from controls. The performance of models decreased significantly when we trained our CNN using activations from task-irrelevant brain regions or from a brain network that is irrelevant to fear. Our results suggest that neuroimaging data analytics of task-induced brain activations within the fear network might provide novel prospects for development of brain-based psychiatric diagnosis.

## Introduction

Nearly all medical fields rely on biological metrics that help clinicians with accurate diagnoses and monitoring of treatment outcomes. Psychiatry is one exception where both diagnosis and treatment outcome are assessed based on the clinician’s observations and patient reporting. Can we develop neurobiologically based approaches to assist with, or improve, efficacy and accuracy of diagnosis and treatment in psychiatry? One way to begin to answer this question is to apply machine learning approaches to study neural pattern of activations within a well-established task and well-studied psychopathologies. We have learned that the amygdala, hippocampus, regions within the medial prefrontal cortex, and the insular cortex are key components of a network that mediates fear, arousal, threat-detection, and regulating responses to fearful and conditioned stimuli^1–6^. Henceforth in this article we refer to the aggregate of these brain regions as the “fear network”. Dysfunction of this fear network has been observed in populations with post-traumatic stress disorder (PTSD)^7–11^ and anxiety disorders^12–16^ using fear conditioning and extinction paradigms. These data have informed us about the mechanisms involved in the acquisition and extinction of conditioned fear in healthy controls and the relevance of fear network abnormalities to the pathophysiology of anxiety and PTSD. This knowledge base makes the study of fear-induced activations within PTSD and anxiety disorders one ideal starting point to exploring brain-based approaches to classify psychopathology.

Machine learning approaches have recently generated growing interest in medicine and psychiatry, with applications in data-driven biomarker diagnoses^17–19^. Some machine learning-empowered studies have shown the possibility to diagnose anxiety-related disorders using functional neuroimaging data^20–23^. However, most of these preliminary studies relied on a relatively small sample size (*N* < 100) as reviewed in a recent study^24^, which may suffer from overfitting problems^25^, and therefore compromise the reliability and generalizability of their predictive power^26,27^. Furthermore, most existing machine learning-based diagnosis studies used brain activation features derived from resting-state functional magnetic resonance imaging (rs-fMRI). Previous studies have suggested that many factors, such as recent experiences and mind wandering, may alter rs-fMRI measures^28,29^. Therefore, patterns derived from rs-fMRI likely reflect influences from arousal, attention, and conscious thought. In contrast, tasks may require participants to be more engaged, and offer an opportunity to manipulate or induce brain state into relevant circuitry^30,31^. Therefore, task fMRI may be better in capturing individual differences in cognition and behavior that might be of relevance to the psychiatric disorders being studied.

In the present study, we used machine learning algorithms and functional activations across 10 brain regions within the fear network and across all training phases in our fear conditioning and extinction paradigm and across a heterogeneous patient population. We asked two specific questions: (1) can we distinguish an anxious or trauma-exposed brain from healthy control’s brain, and (2) how essential the activations of the nodes within the fear network (task-specific activations) are in this discrimination? With a relatively large dataset, we demonstrate that a neural network-based approach can distinguish anxious or trauma-exposed brains from matched controls. We further conducted several specificity analyses to demonstrate that the fear network had significantly stronger predictive power compared to other brain regions. Classification analyses to distinguish subtypes of anxiety or PTSD were not conducted due to small sample size. In summary, we have demonstrated that fear-induced neuroimaging data analytics can reliably distinguish anxious and trauma-exposed individuals from controls.

## Materials and methods

### Participants

**Fig. 1:**
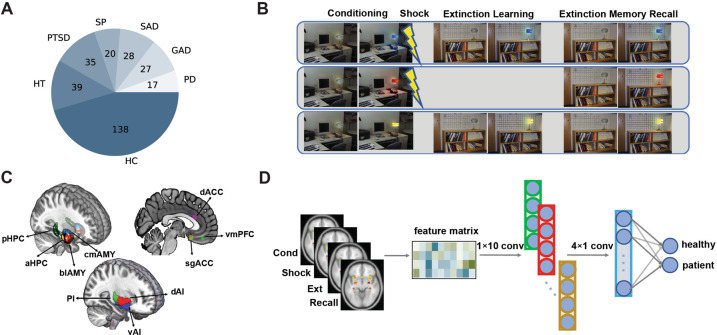
Overview of data, experimental paradigm, and neural network classifier. **A** Summary of participants in each category. HC healthy control, HT trauma-exposed healthy, PTSD post-traumatic stress disorder, SP specific phobia, SAD social anxiety disorder, GAD generalized anxiety disorder, PD panic disorder. **B** The fear conditioning and extinction paradigm consisting of four phases: fear conditioning (‘Cond’), unconditioned shock (‘Shock’), extinction learning (‘Ext’), and extinction memory recall (‘Recall’). **C** Ten target brain regions within the fear network. **D** Architecture of a three-layer convolutional neural network.

This cross-sectional study of 304 adults (111 men, 193 women) aged 18–65 years included 92 anxiety patients, 74 trauma-exposed individuals (35 of which with PTSD diagnosis), and 138 matched controls (Fig. 1A). Among the anxiety group, there were 24 patients diagnosed with generalized anxiety disorder (GAD), 17 panic disorder (PD) patients, 31 social anxiety disorder (SAD) patients, and 20 specific phobia (SP) patients. Data from this sample have been published elsewhere focusing on the neural mechanisms of fear conditioning and extinction within PTSD and anxiety disorders^8,12,32^. Specific and detailed criteria pertaining to each patient population has been detailed in these previous publications. For review of exclusion criteria and a description of the study sample, see Methods section in the Supplemental Material. Demographic characteristics of this sample was listed in Table S1. This study was approved by the institutional review board of Partners HealthCare. Written informed consent was obtained from all participants.

### Experimental procedure

All subjects underwent the same two-day fear conditioning and extinction paradigm in a fMRI scanner (Fig. 1B) which is described in details in our prior publications^8,9,33–35^. On day 1, fear conditioning occurred, during which 2 cues were paired with a shock (CS+, 62.5% reinforced) and 1 cue was not paired with a shock (CS−). This was followed by extinction learning, where 1 CS + and the CS- were presented without shock. On day 2, extinction memory recall was tested with all 3 cues, including the extinguished CS + (CS + E), the unextinguished CS + (CS + U), and the CS− (details are provided in the Methods section in the Supplemental material).

### Data processing

Neuroimaging data were preprocessed as previously described^9,12,32,36^. We extracted brain activation features across four phases: fear conditioning (‘Cond’), unconditioned response to the shock (‘Shock’), extinction learning (‘Ext’), and extinction recall (‘Recall’) from first-level contrast images. The contrasts used to define activation of each phase were: onsets of CS + vs. CS− for ‘Cond’ (all trials of each CS), offsets of reinforced CS + vs. unreinforced CS + for ‘Shock’ (all trials of each CS), onsets of CS + vs. CS− for ‘Ext’ (the last 4 trials of each CS), and onsets of CS + E vs. CS + U for ‘Recall’ (the first 4 trials of each CS). Based on previous studies, we focused our analysis on mean activations from 10 predefined regions of interest (ROIs) which are deemed the key components of fear network (Fig. 1C): centromedial amygdala (cmAMY), basolateral amygdala (blAMY), bilateral anterior hippocampus (aHPC), bilateral posterior hippocampus (pHPC), subgenual anterior cingulate cortex (sgACC), ventromedial prefrontal cortex (vmPFC), dorsal anterior cingulate cortex (dACC), dorsal anterior insula (dAI), ventral anterior insula (vAI), and posterior insula (PI). The way in which each of these brain regions is defined is described in the Supplemental material. And the distributions of these brain activations were shown in Figure S1. We divided the amygdala and the hippocampus into sub-regions because both animal and human neuroimaging studies have suggested that blAMY and cmAMY might have distinct functional roles in fear processing: the blAMY is more related to fear-related associative learning, whereas cmAMY is more related to fear expression^3,37,38^. Similarly, evidence suggests a different functionality of anterior vs. posterior areas of the hippocampus^39–41^. We focused on the fear network in our study since these regions have been implicated in fear processing, and prior studies have consistently reported that abnormal activations of these regions are related to the pathophysiology of anxiety and PTSD^1,3,10,42,43^. Although there may be more regions involved in fear processing, we did not try to include all of them, because a large number of features may lead to the overfitting problem in machine learning^25^, especially when the sample size is not large^44^.

### Machine learning analyses

We applied machine-learning classifiers to discriminate anxious brains from non-anxious brains, or trauma-exposed brains from controls. We constructed a convolutional neural network (CNN) for the classification (Fig. 1D). The input of the CNN is the fear-induced fMRI activations from the 10 ROIs across 4 phases. The output of the CNN is the prediction score ranging from 0 to 1, which is the probability that the subject belongs to the anxious (or trauma) group^25^. We assessed the classifier generalizability using a 5-fold stratified cross-validation (repeated for 100 times to increase stability), reported the area under receiver operating characteristic curve (AUC)^17^. We used a non-parametric permutation test to determine the statistical significance of the classification results. We assessed the discriminative importance of features by doing classification with the corresponding features removed.

We also conducted a cross-subtype classification analysis. Specifically, we excluded subjects from a specific type of anxiety disorder (e.g. GAD) and paired them with a matched number of randomly selected controls as the testing data and used the remaining data as the training data.

We conducted three different specificity analyses to examine the specificity of the fear network in the discrimination. First, we randomly selected ten brain regions from the whole brain and used their brain activations for classification. Second, we randomly selected 10 regions from the somatomotor network for the classification. Third, we randomly replaced *N* brain regions from the fear network with *N* (ranges from 1 to 9) randomly selected brain regions outside of fear network.

We compared the CNN with several classical classifiers, including support vector machine with linear kernel (SVM), SVM with Gaussian radial basis function (RBF) kernel (SVM-rbf), Gaussian process classifier with RBF kernel (GP), random forest (RF), and logistic regression with L2 regularization (LR). We also investigated the impact of sample size on the classification (Methods of the Supplemental material).

## Results

### Discriminating anxious from non-anxious brains

**Fig. 2:**
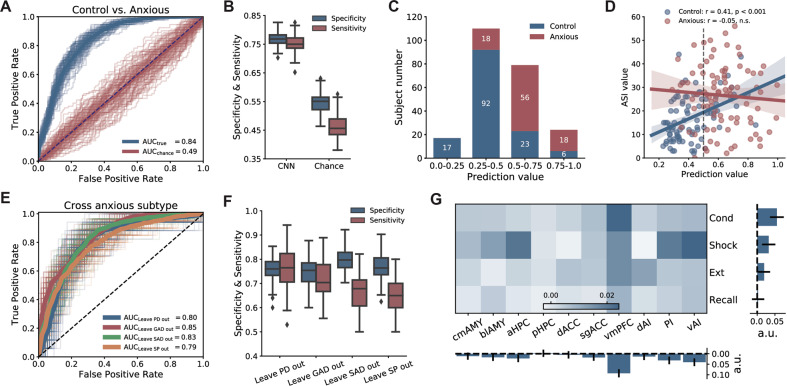
Performance for discriminating anxious brains with various types of anxiety disorder. **A** ROC curves obtained using 5-fold cross-validation. As a comparison, the ROC curves derived from shuffled data and the chance-level AUC are shown. **B** Box plots of specificity and sensitivity. The whiskers extend to the most extreme data points not considered outliers, and the outliers are labeled as ‘♦‘. **C** Empirical distribution of prediction scores. **D** The prediction score positively correlated with the anxiety sensitivity index (ASI) for the control group (*r* = 0.41, *p* = 7.4e-5), but at the chance level for anxious brains (*r* = −0.05, *p* = 0.65). **E** Generalization of cross-subtypes in anxiety disorders (e.g., PD, GAD, SAD, or SP) was assessed by classification analysis. **F** Box plots of specificity and sensitivity in cross-subtype classification. **G** The relative importance of 40 brain activation features assessed by their contributions. A greater positive coefficient (a.u.) implied more importance.

The CNN revealed a mean AUC of 0.84 ± 0.01, which was significantly higher than the chance level (0.49 ± 0.05, *p* < 0.001, Fig. 2A). Based on the CNN’s prediction score, we classified subjects into anxious or non-anxious brains, with 0.75 ± 0.03 sensitivity and 0.77 ± 0.02 specificity (Fig. 2B). Classification performance was similar across both males and females (odds ratio = 0.61, *p* = 0.17) in classification performance. In the cross-subtype classification analysis, the derived mean AUCs were similar across four anxiety disorder subtypes (leave PD: 0.80 ± 0.04, leave GAD: 0.85 ± 0.03, leave SAD: 0.83 ± 0.03, leave SP: 0.79 ± 0.02, all *p* < 0.001, Fig. 2E, F).

### Correlations between anxiety measures and prediction score

To examine the distribution of prediction scores derived from the CNN output, we split them into four percentiles (0.0–0.25, 0.25–0.50, 0.50–0.75, 0.75–1.0). The accuracy in classifying anxious participants increased as prediction scores increased; for example, classification accuracy increased from 76.9% for prediction scores located in 0.25–0.75 bins compared to 87.5% for prediction score located in 0–0.25 or 0.75–1 (Fig. 2C). In the control group, the prediction score was positively correlated with the score on the anxiety sensitivity index (ASI; *r* = 0.41, 95% CI, 0.22–0.57, *p* < 0.001, Fig. 2D). In the anxious group, there was no significant association between the prediction score and the ASI score (*r* = −0.05, 95% CI, −0.27 to 0.15, *p* = 0.65, Fig. 2D), this correlation value was significantly lower than that for the control group (Δ*r* = 0.46, 95% CI, 0.18–0.72, *p* < 0.001).

### Feature contribution for the classification

We quantified the contribution of different features in diagnosing anxious brains by removing a specific type of features from the data and re-assessed the classification performance. In the presence of a missing feature, we fed the CNN with a constant, which was equal to the mean activation of that feature across all subjects. First, removing a single feature yield a relatively small AUC decrease (range from 0 to 0.02 for different feature). Second, comparing to other phases, removing features from the fear conditioning phase lead to larger AUC decrease (decrease: 0.06). Third, comparing to other ROIs, removing features across four phases from the vmPFC led to the largest AUC decrease (decreased value: 0.08). Overall, these feature ranking analyses suggest that the activations from the fear conditioning phase and the vmPFC contributed the most in distinguishing anxious brains from controls (Fig. 2G).

### Discriminating trauma-exposed brains from controls

**Fig. 3:**
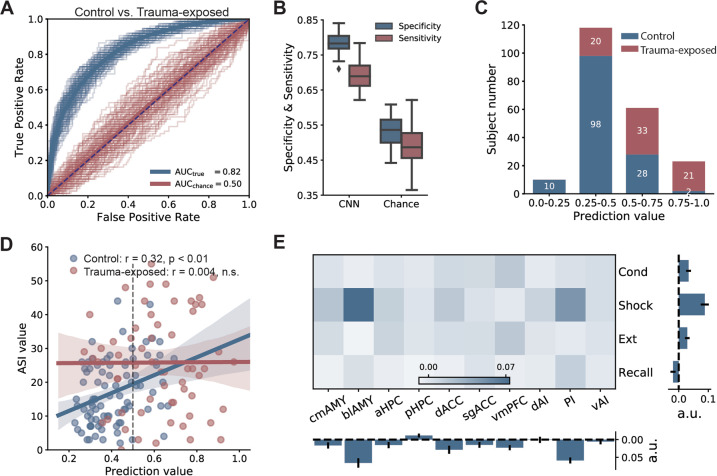
Performance for discriminating trauma-exposed brains from controls. **A** ROC curves derived from 5-fold cross-validation. **B** Box plots of specificity and sensitivity. **C** Empirical distribution of prediction scores. **D** The prediction score positively correlated with ASI for the control group (Spearman *r* = 0.32, *p* = 0.0024), but at the chance level for trauma-exposed group (Spearman *r* = 0.004, *p* = 0.97). **E** The relative importance of 40 brain activation features assessed by their contributions.

We employed the identical CNN architecture, but retrained the network parameters to discriminate trauma-exposed individuals from controls. The mean AUC was 0.82 ± 0.01 (*p* < 0.001, Fig. 3A). Overall, the prediction scores from the trauma-exposed individuals were predominately found in the higher percentile (Fig. 3C). The prediction score of the control group was significantly correlated with the ASI (*r* = 0.32, 95% CI, 0.13–0.50, *p* = 0.002; Fig. 3D). In contrast, there was no significant association between the prediction score and the ASI for the trauma-exposed group (*r* = 0.004, 95% CI, −0.24 to 0.26, *p* = 0.97; Fig. 3D). Since the difference between these two correlations was not significant (Δ*r* = 0.32, 95% CI, −0.01 to 0.61, *p* = 0.06), these results should be interpreted with caution due to a smaller sample size used in model training. For feature importance, brain activations from the shock phase contributed higher than other phases to the classification. Removing these features would decrease the AUC by more than 0.05 (Fig. 3E). For the ROI features, removing the blAMY or PI caused a large decrease in AUCs (both larger than 0.05). Importantly, the derived feature importance map was different from the one derived earlier (Fig. 3E vs. Fig. 2G), suggesting that trauma and anxiety may differentially modulate the fear network in a task-specific manner. Furthermore, we examined whether different psychopathologies can be discriminated from one another using machine learning. Specifically, we ran a similar classification analysis to discriminate anxious from trauma-exposed brains. The CNN obtained a mean AUC of 0.80 ± 0.02, which was higher than other compared classifiers (Fig. S2 in the Supplemental material).

### Specificity analysis using randomly selected brain regions

**Fig. 4:**
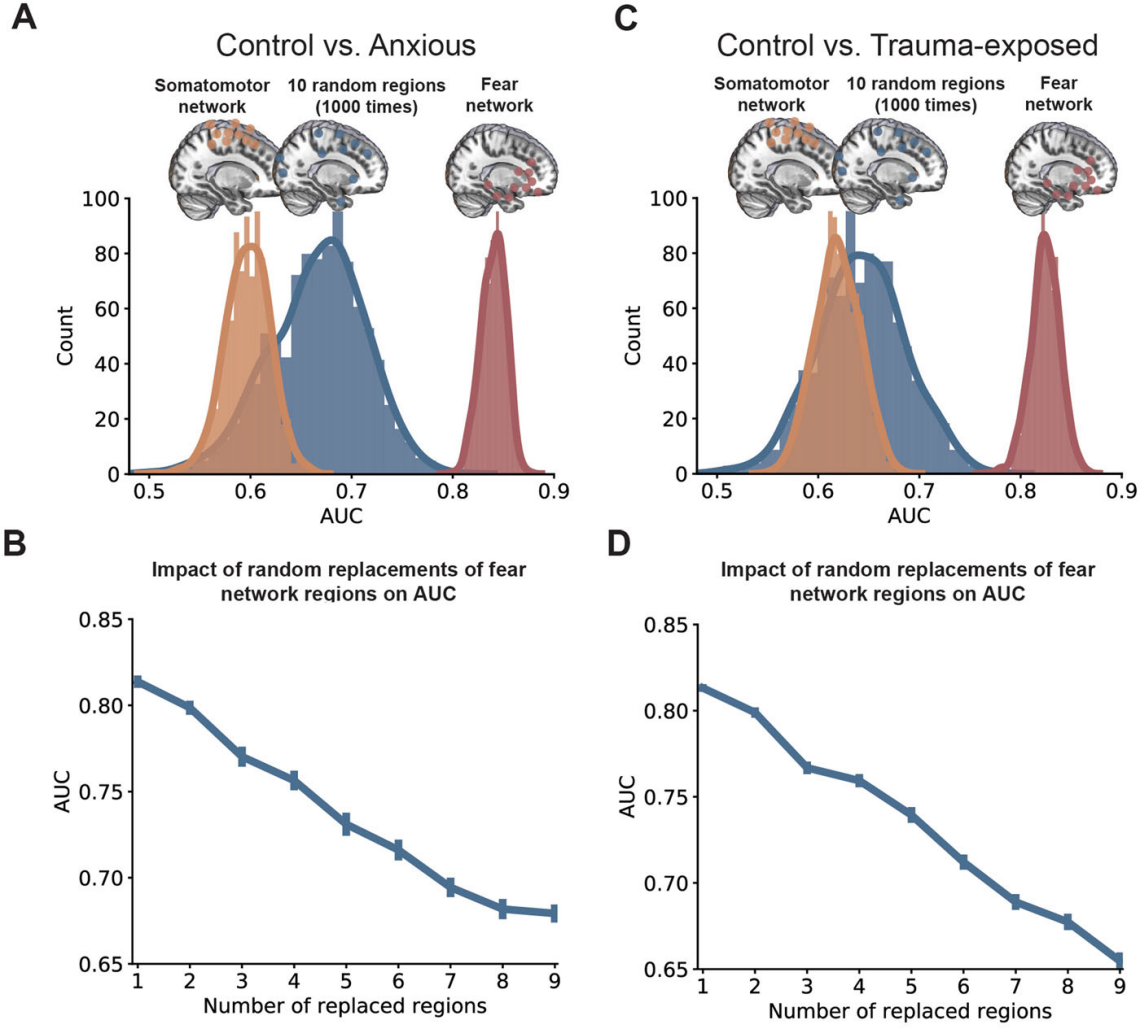
Specificity analysis of the fear network in classification. **A** Distribution of AUCs based on brain activations within the 10-node fear network, or 10 regions within the somotomotor network, or 10 randomly selected brain regions for Controls vs. Anxious. **B** Mean AUCs were shown when a specific number of regions within the fear network were replaced by a mathced number of randomly selected brain regions for Controls vs.Anxious. **C**, **D** Corresponding analyses for Controls vs. Trauma-exposed.

We conducted three follow-up specificity analyses to ask how critical are the activations of the fear network contributed to the classification. First, when using activations of 10 randomly selected brain, the obtained AUCs (mean AUC: 0.67 ± 0.05) were significantly lower than the AUC derived from the fear network (ΔAUC: 0.17 ± 0.05, *p* < 0.001; Fig. 4A). Second, the 10 brain regions from a somatomotor network also led to significant degradation of the AUC (mean AUC: 0.60 ± 0.02) when compared to the target fear network (ΔAUC: 0.24 ± 0.03, *p* < 0.001; Fig. 4A). Third, replacing *N* (*N* = 1–9) of 10 fear network brain regions with *N* other randomly selected brain regions caused a monotonic decrease in AUCs with increasing *N* (Fig. 4B). The correlation between the prediction score and the ASI for controls also decreased when we switched from the fear network to other regions (Fig. S3 in the Supplemental material). Similar results were obtained when comparing controls with trauma-exposed brains, where AUCs obtained using randomly selected brain regions (mean AUC: 0.64 ± 0.04) or activations from the somatomotor network (mean AUC: 0.62 ± 0.02) are significantly smaller than the AUC derived from the fear network (ΔAUC: 0.18 ± 0.05, ΔAUC: 0.21 ± 0.03, both *p* < 0.001; Fig. 4C). There was a monotonic decrease in AUC when an increasing number of fear network nodes was replaced with activations from randomly selected brain regions (Fig. 4D). We conducted an exploratory analysis by incorporating feature selection into the cross-validation procedure. We selected 10 other fear-related regions based on a meta-analysis study^2^, and conducted feature selection within the cross-validation procedure (see Supplemental Material for more details). We obtained similar results as in our main analysis, with an AUC of 0.79 ± 0.02 for anxiety vs. control, AUC of 0.77 ± 0.02 for trauma-exposed vs. control (Fig. S4). Notably, brain regions from the fear network were frequently selected across the cross-validation procedure (Fig. S5). We also found that using these 10 regions resulted in degraded performance than the fear network (Fig. S6). Overall, these results suggested that the selected fear network contains critical information for distinguishing anxious/trauma-exposed brains from controls.

### Comparison of classifiers and impact of sample size

**Fig. 5:**
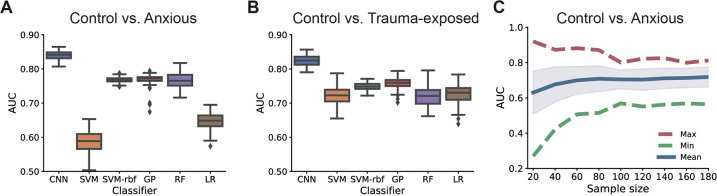
AUCs derived from different classifiers and different sample size. **A** The cross-validated AUC statistic derived from our proposed CNN was better than the other five tested classifiers in Control vs. Anxious and **B** Control vs. Trauma-exposed. **C** Small sample size resulted in large standard deviations of classification accuracies. CNN convolutional neural network, SVM support vector machine with linear kernel, SVM-rbf SVM with Gaussian radial basis function (RBF) kernel, GP Gaussian process classifier with RBF kernel, RF random forest, LR logistic regression with L2 regularization. Box plots show the AUC statistics derived from 100 random selections in 5-fold cross-validation.

Comparing with several standard machine-learning classifiers, the CNN yielded a better performance (Fig. 5A, B), suggesting that the CNN can potentially extract higher-order nonlinear features that were beyond the power of other nonlinear classifiers. We also investigated the impact of sample size on the classification performance, by randomly selected a subset of subjects for cross-validation (Fig. 5C). The AUCs exhibited an increasing degree of variability when the sample sizes were decreased. For instance, when the sample size was reduced to 20, the maximum AUC was higher than 0.9, whereas the mean AUC derived from 100 sampling populations was ~0.6.

## Discussion

Here we investigated whether fear-induced brain activations^1–3^ can be used to identify anxious or trauma-exposed brains from those that are not. We examined brain activations of more than 300 subjects that underwent a fear conditioning and extinction task. The combination of deep learning strategy (i.e., CNN) and brain activations of the fear network across 4 learning phases enabled us to distinguish anxious and trauma-exposed brains from controls. We further conducted a series of analyses to show that task-driven activations within the fear network provide specific and significant discriminative information compared to task-irrelevant brain regions as well as compared to a brain network not critical for emotion regulation.

Our analyses focused on activations from 10 specific brain regions in building our machine learning model. The selection of these regions was based on accumulating evidence showing that their activations are relevant to emotion expression and regulation, fear learning and extinction, and are dysfunctional in psychopathology^1–3,13,45^. Our ROI-based analyses are novel and distinct from most previous machine learning studies that have relied on whole-brain activations^20,22,46^ or on functional networks estimated from resting-state fMRI^20,47–52^. With collected features from the whole brain, it might be challenging to interpret the results of the obtained models in these studies^53^. In contrast, by concentrating on task-induced activations within the fear network, the CNN model is restricted to linking brain activations in fear learning and extinction with psychiatric states. Notably, when we switched from the fear network to randomly selected brain regions or a brain network not critical for fear learning or extinction, the discriminative performance significantly decreased (Fig. 4). These results highlight the specificity of the fear network and its activation during all experimental phases to further our understanding of the psychopathology underlying PTSD and anxiety disorders.

Our study focused on fear-related task-induced activations, which we believe offers a significant advantage over most of previous resting-state-based studies. Specifically, in a recent anxiety-related machine learning literature survey, only 2 of the 23 reviewed studies relied on task-based fMRI data^24^. Increasing evidence suggests that participants’ specific traits were better predicted when the subjects attended the tasks^31,54^. Since the fear conditioning and extinction paradigm is highly relevant to the pathological of anxiety- and fear-based disorders^36,45,55–58^, and numerous studies have observed and replicated dysregulated neural activations during emotional regulation in fear- and anxiety-related disorders^1,3,59^, it is natural to expect the fear-induced activations would serve as a more specific neural signature in classifying psychopathology. However, we note that both task and rs-fMRI have their pros and cons. For example, rs-fMRI is more convenient in data acquisition, especially in clinical settings. The fear conditioning and extinction paradigm lasts 2 days, which may increase dropout rate of participants. For a more detailed comparison of rs-fMRI and task fMRI, please see Daliri and Behroozi^60^.

One interesting finding obtained from our CNN model is that while activations from all 10 brain regions across all phases were important for our classifications, there were some differences between the prediction of the anxious and trauma-exposed brains. Activations within the conditioning phase of our experiment provided more robust contributions to predicting patients with anxiety disorders. Activations related to the shock response during fear conditioning, on the other hand, had the most robust contributions to our models in predicting the trauma-exposed individuals. These results show that while activations within the network during extinction learning and extinction recall were important, more robust contributions came from experimental phases associated with fear acquisition and response to the aversive cues. These results are consistent with prior studies showing the importance of stress responses and variance in cortisol levels to the pathophysiology of PTSD and anxiety disorders^42^.

The observed significant association between the prediction score and the anxiety sensitivity index (ASI) in the control group supports the idea that the interactive functional activation of the interrogated brain regions during threat conditioning and its extinction might contribute to anxiety. It is, however, intriguing that this association was absent in the patient group. The lack of correlation within the anxiety group is unlikely to be related to a ‘ceiling effect’, since the ASI values and prediction scores for anxiety/trauma-exposed groups were broadly distributed, which would have allowed the detection of an association. A possible reason is that the control group was more homogeneous than the anxious/trauma-exposed groups. First, the anxious/trauma-exposed groups included participants with different psychiatric diagnoses, such as general anxiety disorder and social anxiety disorder. Second, recent neuroimaging studies have shown that the control group is more homogeneous than groups with psychiatric disorders^61,62^. The homogeneity of the control group led to having similar small prediction scores, except those that were atypical, i.e., with higher ASI values. As we can see from Fig. 2D, individuals with lower ASI values were well clustered with low prediction scores.

We employed the same architecture of the CNN in two classification tasks (anxious vs. controls and trauma-exposed vs. controls). Both tasks have resulted in AUCs >0.8 (Figs. 2A and 3A), with an adequate tradeoff between sensitivity and specificity (Figs. 2B and 3B), suggesting generalizability of the CNN classifier. Furthermore, individuals from four distinct anxiety disorders were included in this study, making our dataset highly heterogeneous. To our knowledge, no study has investigated the possibility to generalize classification across subtypes of anxiety disorders. Most studies have either recruited individuals from one particular anxiety disorder or treated subtypes of anxiety disorders separately^22,63^. In our cross-subtype classification analysis, the obtained AUCs were ~0.8 when a specific subtype of individuals was left out as the testing data (Fig. 2E), which achieved similar accuracy as when all anxiety patients were included in the analysis. This cross-subtype classification results support the Research Domain Criteria (RDoC) approach^64^. We have recently published a study showing that there are advantages to use the RDoC approach in learning about the psychopathology of anxiety disorders^32^. The results from this study require further validation across a larger sample of patients with PTSD and anxiety disorders.

Another strength in our results is the sample size examined. Concerns have been raised regarding the reliability and generalizability of prediction studies with small sample size^17,26^. Here, functional neuroimaging of more than 300 individuals were examined to explore reliable psychiatric biomarkers. Our sample size is substantially larger than previous anxiety- and fear-related disorder diagnosis studies where the sample sizes were mostly fewer than 100 as reviewed in a recent study^24^. Although there are neuroimaging biomarker studies with large sample sizes for other disorders such as schizophrenia^65^ and autism^66^, they are based on resting-state fMRI. A small sample size can lead to biased predictive accuracy, especially when the leave-one-out cross-validation procedure was employed^17^. Our results show that variance of accuracy increased as sample size is decreased (Fig. 5C). These results are well-aligned with previous studies suggesting that performances derived from small samples are inflated^26^. We have examined the fear network activations using the recommended 5-fold cross-validation procedure^17^ in two classification tasks, which increased the reliability of the results. Future studies with significantly larger sample sizes should be conducted to test the generalizability of our data across larger populations. And perhaps with larger sample size, additional analyses could be conducted to test the possibility of distinguishing subtypes of anxiety disorders from one another and PTSD from trauma-exposed non-PTSD. In our exploratory analysis, we found that anxious and trauma-exposed brains can be discriminated from each other using activations of the fear network (Fig. S2). Future analyses can be conducted to further explore how the fear network is modulated across different psychopathologies.

It is important to note that there exist skeptical views within the field regarding the clinical utility of, or the need for, neurobiological markers for anxiety and trauma. The argument against the biomarkers here is that anxiety and trauma-related symptoms are easy to diagnose and the tests to be conducted for their diagnosis would not be needed, would be costly, and time consuming. We argue, however, that there is a clinical value as some patients may not fully disclose all symptoms and others may wish to have a biological explanation for why they feel the way they do. Another challenge to establish neurobiological markers for psychiatric disorders is that current methods for diagnosis are largely based on self-report data from the patients. These self-report data are very subjective to the person experiencing the symptoms and cause a high degree of variability across subjects, even within a given diagnostic group. The result of the large variance in how patients experience their symptoms often leads to absence of meaningful or significant correlations between symptoms and psycho-behavioral indices from experimental tasks, even if they are hypothesized to measure related cognitive and emotional processes^67,68^. Therefore, the ideal ‘diagnosis’ to establish a ‘biological marker’ might be very broad and lacks clear boundaries, and such will be a major limitation to experimental effort. In our study, we used the clinical labels in our supervised case–control classification analyses similar to the literature. In this paper, we provide more of a conceptional exploration in the direction towards the neurobiological biomarker development. Our results suggested that the activations of the fear network are likely to provide critical information to distinguish anxious and trauma-exposed brains from those that are not. The insights gained from this study could be subsequently applied to follow-up explorations in this domain.

There are some limitations to the current study. First, we used cross-validation to assess model performance. An independent test set should be used in the future to further assess the generalizability of the proposed model. Second, the fear conditioning and extinction paradigm lasts 2 days, which may make data collection more difficult than rs-fMRI. Third, we used activations of the fear network for the classification in this study, which may overlook the functional connectivity between brain regions. Connectivity analyses that were widely used in rs-fMRI may be incorporated as additional features to further improve the classification performance.

In conclusion, we report data showing that a deep learning-empowered data analytic approach can distinguish anxious and trauma-exposed brains from controls using fear-induced brain activations. The fear network (task-specific) activations exhibited more discriminative information than activations obtained from other brain regions. Our results suggest that fear-induced brain activations within the fear network may serve as potential specific biomarkers for psychiatric diagnosis.

## Supplementary information

Supplemental material
