# Supplementary material for: Fear-induced brain activations distinguish anxious and trauma-exposed brains
Source: Transl Psychiatry. 2021 Jan 13;11:46. doi: 10.1038/s41398-020-01193-7 (PMC7806917; doi:10.1038/s41398-020-01193-7)
Supplement: Supplementary file 1 — Supplemental material [file 41398_2020_1193_MOESM1_ESM.docx]

**Supplementary material**

Zhenfu Wen, Marie-France Marin, Jennifer Urbano Blackford, Zhe S. Chen, Mohammed R. Milad. **Fear-induced brain activations distinguish anxious and trauma-exposed brains.**

**Methods**

**Participants**

All data used in this manuscript had been used for prior studies focusing on group differences within fear extinction and had been previously published^1–4^. The demographics of all participants had been described and published in these studies. A total of 304 subjects (111 men, 193 women) participated in the current study: 92 anxiety patients, 74 trauma-exposed individuals (35 of which with PTSD diagnosis), and 138 matched controls without diagnosed anxiety or trauma. Among the anxiety group, there were 24 patients of generalized anxiety disorder (GAD), 17 patients of panic disorder (PD), and 31 patients of social anxiety disorder (SAD) and 20 patients of specific phobia (SP). The following exclusion criteria were used during participant recruitment: history of seizures, current substance abuse or dependence, metal implants, pregnancy, breastfeeding, or positive urine toxicology screen for drugs of abuse. Biographic information of this sample was listed in **Table S1**. This study was approved by the Institute Review Board (IRB) of the Massachusetts General Hospital, Harvard Medical School.

**Experimental procedure**

All subjects underwent a two-day fear conditioning and extinction paradigm in a fMRI scanner. The detailed paradigm has been described previously and are briefly described below^1,5–8^. The paradigm consisted of several stages, and the trial structure was identical across different stages. Each trial started with a blank screen lasting 12-18 s (mean: 15 s), followed by a picture of a room (either a library or an office) with an unlit lamp for 3 s (context) after which the lamp turned on to blue, red or yellow and lasted for 6 s (conditioned stimulus, CS). On the first day, participants were instructed to select their level of electric stimulation to be used during the experiment, so that the stimulation level was highly annoying yet non-painful. Electrical stimulation was delivered through electrodes that were attached to the index finger and middle finger of the participant’s dominant (right) hand. The paradigm then started with a habituation stage in which all paradigm images were presented to the participant, without any electrical stimulation. Following the habituation, fear conditioning occurred in one context (e.g., the ‘office’ picture) where two of the colored lamps (e.g., blue and red, CS+) and partially reinforced (62.5% reinforcement rate) with a mild electric shock (500 ms, occurring at the offset of CS presentation) and the other colored lamp (e.g., yellow) were never paired with the shock (CS-). Fear conditioning consisted of a total of 32 trials (8 presentations for each of the two CS+s and 16 presentations of CS-). Following this, extinction learning occurred in a different context (e.g., the library), where one of CS+ was presented 16 times without any shocks (CS+ extinguished; CS+E) intermixed with 16 presentations of CS-. On the next day, extinction memory recall was tested in the context of extinction learning (e.g., the ‘library’ picture), where the three colored lamps were presented (8 presentations of the extinguished CS+E, 8 presentations of the unextinguished CS+ (CS+U) along with 16 presentations of CS-). The order of stimulus presentation was pseudo-random for all stages of the paradigm.

**MRI acquisition and preprocessing**

Human neuroimaging data were acquired in a Trio 3.0 Tesla whole-body MRI scanner (Siemens Medical Systems, Iselin, NJ) using a 32-channel head coil. Functional data were acquired using a T2*-weighted echo-planar imaging (EPI) pulse sequence (TR: 2.56 s, TE: 30 ms, slice number: 48, voxel size: 3×3×3 mm). Anatomical images were acquired using a T1-weighted MPRAGE pulse sequence (1 mm3 resolution). MATLAB and SPM12 were used for all imaging data analyses. Functional images were corrected for slice timing, realigned, co-registered with the structural image, normalized into MNI space, and smoothed with an 8-mm full width half-maximum Gaussian kernel. Signal drift and biorhythms were modeled using high-pass temporal filtering (128 s) and a first-order autoregressive model. An artifact detection toolbox (ART, http://gablab.mit.edu) was used to identify motion artifacts. Motion outliers detected by ART were used in the first-level analysis as regressors with movement parameters (x, y, z, roll, pitch, and yaw) from the realignment process. Motion regressors generated from the ART-tool were then applied to all first-level analyses.

**Feature extraction**

We extracted brain activation features from first-level contrast images for the classification analyses. During fear conditioning, the 16 CS+ trials were compared to the 16 CS- trials (fear conditioning phase) to capture the conditioned aspect of fear, and the 10 CS+ trials paired with a shock were compared to the 6 omitted shock trials (shock phase) to capture the unconditioned aspect of fear, i.e., the reaction to the aversive stimulus (shock). For fear extinction learning, we compared the last 4 CS+ trials with the last 4 CS- trials (extinction phase) in order to capture the end of the extinction learning process. During extinction recall, we focused on the early phase to avoid a second extinction learning process and we therefore compared the first 4 trials of CS+ E were compared to the first 4 trials of CS+U (recall phase). The definitions of these contrasts were largely based on previous studies^1,3–5,9^.

The mean contrast values were then extracted from ten regions of the fear network, including centromedial amygdala (cmAMY), basolateral amygdala (blAMY), bilateral anterior hippocampus (aHPC), bilateral posterior hippocampus (pHPC), subgenual anterior cingulate cortex (sgACC), ventromedial prefrontal cortex (vmPFC), dorsal anterior cingulate cortex (dACC), dorsal anterior insula (dAI), ventral anterior insula (vAI) and posterior insula (PI). These 10 target regions are largely involved in affect regulation, and the dysfunction of these regions has been observed in populations with post-traumatic stress disorder (PTSD)^1,5,9^ and anxiety disorder^3^. The definitions of these regions were as follows: The masks of dACC, sgACC and vmPFC were created with the use of Neurosynth (neurosynth.org), which is a searchable online automated synthesis of fMRI data. By using the keywords ‘fear conditioning’, we identified clusters within 248 studies with estimated peaks at the following coordinates: dACC (MNI_xyz_ = 0, 14, 28), sgACC (MNI_xyz_ = 0, 16, -18) and vmPFC (MNI_xyz_ = 6, 40, -20) and created a mask with a sphere (radius: 10 mm) for each region. The masks of cmAMY, blAMY were used from^10^, and the aHPC, pHPC masks were obtained from the Anatomical Automatic Labeling Atlas^11^. The dAI, vAI and PI were obtained from a mask identified through cluster analysis^12^.

**Machine-learning discriminatory analysis**

We applied machine-learning classifiers to discriminate anxious (or trauma-exposed) brains from non-anxious (or non-trauma-exposed) brains (“control” group). Motivated from the success of deep learning, we constructed a convolutional neural network (CNN) to map the fear-induced fMRI activations into a binary diagnosis decision.

The CNN consists of a two-layer convolution-layer architecture to extract hierarchical and nonlinear features. The input to the CNN is a 4×10 (phase×region) feature matrix, followed by a 1×10 convolution layer with 24 filters which can be regard as region combination. The resulted maps were fed into a 4×1 convolution layer with 24 filters to combine features from all four experimental phases. Considering a relatively small sample size (by the deep learning standard), we tried to keep our CNN relatively compact in order to minimize the number of layers or parameters. These combined values were then flattened and fed into the output node to classify the subject to anxiety group or healthy group. We used the rectified nonlinearity unit (RELU) as activation function for all hidden layers and used sigmoid activation for the output layer. To alleviate overfitting, a drop-out layer (with 0.5 probability) was added between every two hidden layers, and a L2-norm regularization was added for each filter. We trained the CNN using the Adam optimizer and the binary cross-entropy loss function. We used a learning rate of 0.001. The batch size was set to 8 and the training process stopped after 300 epochs.

We used a 5-fold stratified cross-validation procedure to assess the classification performance of the proposed CNN. First, the dataset was randomly split into five folds, with the percentage of samples for each class preserved in each fold. Next, CNN was trained with four folds of data and tested with the remaining one fold. This procedure was repeated for five times, with each fold of data served as testing data each time. To accommodate the sample imbalance issue, we employed an undersampling procedure for training data^13^. After that, prediction scores across all five folds of testing data were merged to compute the ROC curve and area under the curve (AUC). We used the AUC instead of accuracy as the main assessment criterion, because of the imbalanced sample size between two classes. To enhance the stability of the AUC results, we further repeated this 5-fold cross-validation procedure for 100 times, reported the mean AUC statistic. In addition, we computed the sensitivity and specificity as follows:

Sensitivity = $\frac{\mathrm{TP}}{TP+FN}$, Specificity = $\frac{\mathrm{TN}}{TN+FP}$

where TP, TN, FP and FN denote true positive, true negative, false positive and false negative, respectively.

For comparison, we also used several classical classifiers in discriminatory analysis, including the support vector machine with a linear kernel (SVM), SVM with a Gaussian radial basis function kernel (SVM-rbf), Gaussian process classifier with Gaussian RBF kernel (GP), random forest (RF) and logistic regression with L2 regularization (LR)^14^.

To investigate the impact of sample size on the classification performance, we randomly selected a subset of subjects from the complete dataset and discriminated anxious or trauma-exposed brains from controls based on SVM-rbf classifier. For each sample size ranging from 20 to 180, we repeated 100 times of the sampling operation, and used a 5-fold stratified cross-validation to assess the classification performance.

**Correlation analysis**

The prediction scores derived from the CNN output were correlated with the anxiety measures of participants using spearman correlation. These analyses were separately conducted on anxious/ trauma-exposed brains and controls who provided Anxiety Sensitivity Index (ASI, controls: N=87, anxious/ trauma-exposed: N=89/61), Beck Anxiety Inventory (BAI, controls: N=102, anxious/ trauma-exposed: N=92/63), Beck Depression Inventory (BDI, controls: N=101, anxious/ trauma-exposed: N=83/72) or State-Trait Anxiety Inventory (STAI-I, controls: N=101, anxious/ trauma-exposed: N=88/67) measures.

**Feature importance**

To assess the importance of individual features in their discriminative power, we estimated the individual contribution of each feature, each region or each experimental phase. Specifically, we replaced the corresponding features in the testing data with the mean values estimated from the training data, and conducted testing based on the trained classifier. Replacing features with positive (or negative) contributions to discrimination would result in a decrease (or increase) in AUC. A greater value of positive coefficient implies more importance of the associated feature. The evaluation procedures were as follows: (i) The CNN model was trained with all features from the training data; (ii) For each type of feature, its value within the testing data was first replaced with the mean values estimated from the training data and then fed into the trained CNN for testing; (iii) The feature importance of each feature was ranked by the difference between the AUC estimated using original testing data and the AUC estimated using the data obtained from procedure (ii).

**Specificity analyses**

We conducted three different control analyses to examine the specificity of the fear network in discriminating anxious/ trauma-exposed brains from the control group. First, we randomly selected ten brain regions and used their brain activations for classification. We randomly selected ten MNI coordinates (with zero overlap with the fear network) within the Automated Anatomical Labeling (AAL) template. We created a mask with a sphere (radius: 10 mm) for each region, and then used these ten masks to extract contrast values for classification. We repeated the procedure for 1000 times, each time with ten randomly selected MNI coordinates. Second, we selected ten regions from the somatomotor network for the classification. We used a whole-brain parcellation consisting of 200 cortical regions^15^, and further extracted contrast features from those ten regions of the somatomotor network. We also tried to randomly select ten regions from the visual network for the classification. Third, we randomly replaced N brain regions from the fear network with N randomly selected brain regions outside of fear network. The value of N ranged from 1 to 9. Therefore, a larger N implied that more features from the fear network would be replaced by other regions. At each N, we repeated the replacement for 100 times.

We conducted an exploratory analysis by incorporating feature selection into the cross-validation. Based on the meta-analysis results from a recent paper^16^, we added 10 regions that distinguish CS+/CS- in human fear conditioning and are not involved in the fear network, including supplementary motor area, precuneus, supramarginal gyrus, middle frontal gyrus, precentral gyrus, postcentral gyrus, superior middle frontal gyrus, inferior frontal gyrus, angular gyrus, and cerebellum. For each region, we created a mask with a sphere (radius: 10 mm) around the peak MNI coordinate reported in Table 2S of that paper. First, we extracted features across 4 phases for each of the 20 regions (10 from the fear network, 10 from the meta-analysis). Second, we separately conducted 5-fold cross-validation with features of each regions (using SVM-rbf classifier, on training data), and ranked each region based on their performance. Third, we selected 10 regions with the highest AUCs, and fed their features into the classification. Note that feature selection was only conducted on training data, to prevent circularity. The results obtained from these analyses were comparable to those reported in the original version of the manuscript, with an AUC of 0.79±0.02 for anxiety vs. control, AUC of 0.77±0.02 for trauma-exposed vs. control (**Figure S4**). We further examined the percentage of time each region was selected across the 100 times of 5-fold cross-validation. We found that regions from the fear network were frequently selected across the cross-validation. Seven (Control vs. Anxious) and six regions (Control vs. Trauma-exposed) from the fear network were selected in more than 60% percentage of the time across the cross-validation (**Figure S5**). We also explored the possibility to use the 10 regions derived from the meta-analysis for the classification, but these regions resulted in lower AUCs than the fear network (**Figure S6**).

**Statistical tests**

We used a non-parametric permutation test to determine the statistical significance of the classification results. Labels of subjects were first shuffled, and the same 5-fold stratified cross-validation procedure as described above were repeated for 1000 times to obtain the chance-level AUC distribution. The p-value was then calculated as the fraction of the 1000 permutated AUCs that were at least as extreme as the true AUC.

**Results**

**Mean activation differences between groups**

To determine whether there are differences in the mean activation between the patient group (anxiety or trauma) and matched healthy controls, we conducted a series of Kruskal-Wallis H-tests on the activation of each ROI at each phase. These tests revealed that there were significant differences in the mean activations of shock-vAI (p=0.03, Bonferroni corrected). Post hoc Wilcoxon rank-sum tests confirmed that the mean activations were different between trauma-exposed individuals and controls (p=0.0008) or between anxious brains and controls (p=0.003). No other significant differences were found after multiple comparison correction. We further examined the distribution of brain activations at ten different brain regions and four different task phases. The distribution of brain activations overlapped substantially between groups, with exceptions in their peak and distribution tails (**Figure S1**). We conducted a two-sample Kolmogorov-Smirnov test to compare the empirical distributions between two groups. In comparing controls with anxious brains, we found significant differences in activation distributions of several regions (cond-sgACC: p=0.045, cond-vmPFC: p=0.0001, shock-vmPFC: p=0.037 and recall-vmPFC: 0.012, all p-values were Bonferroni-corrected by multiplying the number of tests, i.e., 40 here). In comparing controls with trauma-exposed brains, we found significant differences in activation distributions of cond-vmPFC (p=0.004), cond-vAI (p=0.019), shock-cmAMY: (p=0.004), shock-dACC (p=0.011) and shock-dAI (p=0.039, all p-values were Bonferroni-corrected). Despite these significant differences, however, it was noted that distributions of different populations largely overlapped. Our results highlight the importance of shifting the focus of prospective research from between-groups mean difference activations within a given brain region to studying the activation of a network of brain regions and also the variance in the distribution of the activations within.

**Discriminating anxious from trauma-exposed brains**

As an exploratory analysis, we also attempted to discriminate anxious from trauma-exposed brains. We retrained parameters of the proposed CNN framework using data of anxious and trauma-exposed individuals. We repeated the 5-fold cross validation 100 times, obtained a mean AUC of 0.80±0.02 (**Figure S2**). We also used other classifiers to do the discrimination. Overall, other classifiers resulted lower AUCs than the CNN, with the random forest classifier performed the best (AUC: 0.69±0.03). These results suggest that anxious brains response differently from trauma-exposed brains in the fear conditioning and extinction paradigm, and the CNN is likely better at extract higher-order nonlinear features for the discrimination than other traditional classifiers.

**Specificity analysis using randomly selected brain regions**

For distinguishing anxious brains from healthy controls, using randomly selected brain regions (mean r: 0.12±0.05) or activations from the somatomotor network (mean r: 0.08±0.09) are smaller than the AUC derived from the fear network (mean r: 0.38±0.04, **Figure S3**). For distinguishing trauma-exposed brains from healthy controls, using randomly selected brain regions (mean r: 0.10±0.06) or activations from the somatomotor network (mean r: 0.06±0.09) are smaller than the AUC derived from the fear network (mean r: 0.28±0.05). There was a monotonic decrease in correlation value when an increasing number of fear network activations was replaced with activations from randomly selected brain regions (**Figure S3**).

**Table S1.** **Demographic characteristics of the sample.**

|  | **HC** | **PD** | **GAD** | **SAD** | **SP** | **Trauma-exposed** |
| --- | --- | --- | --- | --- | --- | --- |
| **Sample size** | 138 | 17 | 24 | 31 | 20 | 74 |
| **Sex (M)** | 44 | 7 | 10 | 9 | 14 | 33 |
| **Age** | 27.9±7.8 | 28.9±9.7 | 30.7±10.7 | 24.8±10.8 | 32.7±9.9 | 33.5±12.8 |
| **Years of education** | 16.4±2.0 | 15.5±1.9 | 16.8±2.2 | 14.8±2.2 | 16.0±3.2 | 15.4±3.7 |
| **ASI** | 17.0±9.5 | 31.4±10.6 | 26.5±11.4 | 29.1±14.5 | 19.0±15.0 | 25.8±15.0 |
| **BAI** | 2.6±3.0 | 21.7±10.7 | 20.3±11.1 | 19.7±11.4 | 17.3±16.9 | 10.9±12.5 |
| **BDI** | 2.0±3.8 | 14.5±11.9 | 17.0±10.2 | 18.7±11.8 | 10.5±12.0 | 9.4±11.3 |
| **STAI-T** | 33.7±8.3 | 52.9±9.4 | 55.3±7.1 | 52.2±8.9 | 40.1±9.7 | 40.4±14.5 |

**Figure S1. Empirical distributions of brain activations during four experimental phases.**

Brain regions including: cmAMY, blAMY, aHPC, Phpc, sgACC, dACC, vmPFC, dAI, PI, vAI (marked by crossing dots). Three subject groups (control, anxious brain, and trauma-exposed brain) are shown by three distinct colors. Symbol * represents statistical significance (p<0.05) when comparing controls with anxious brains, or comparing controls with trauma-exposed brains.


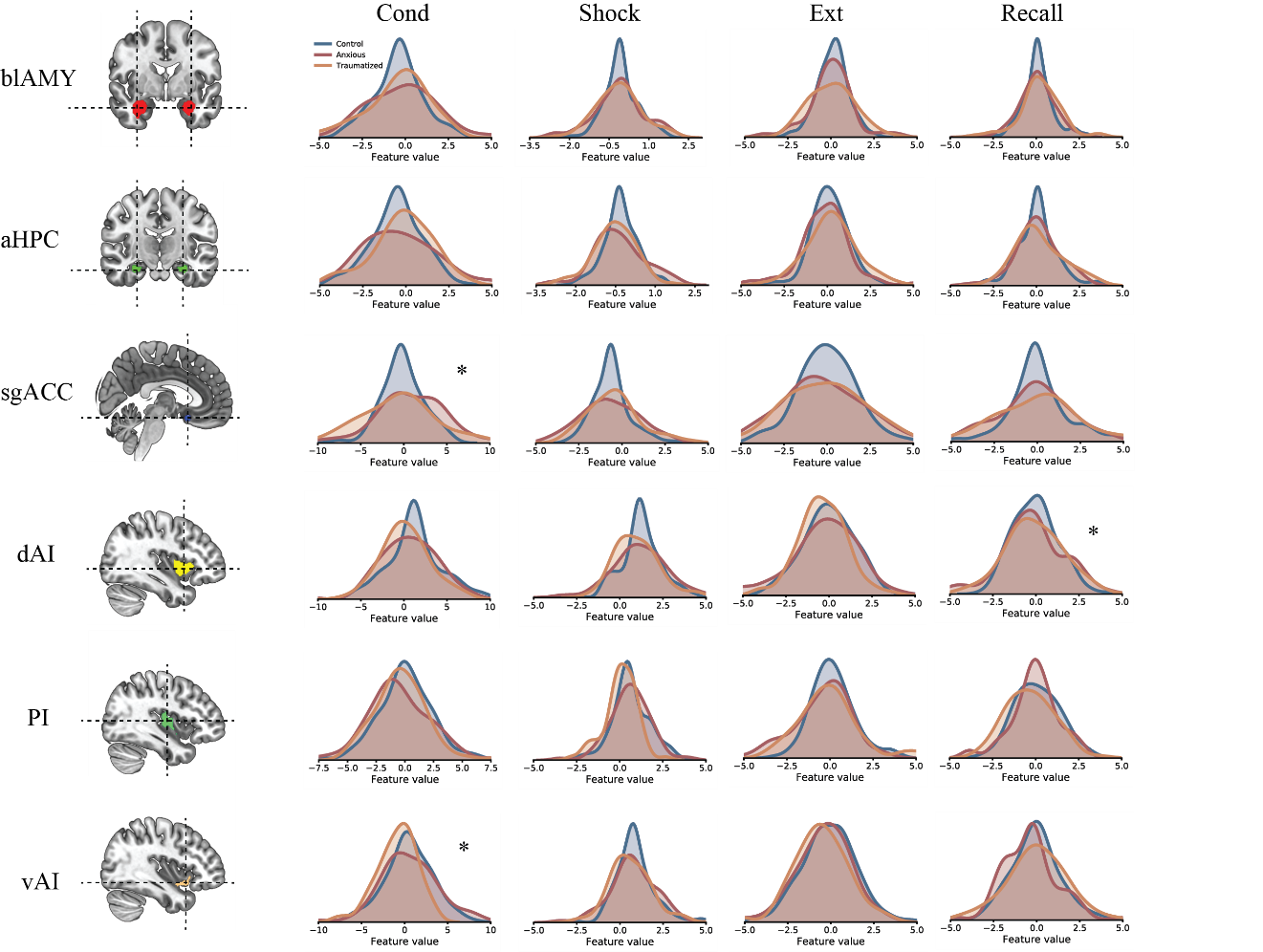


**Figure S2. Discriminating anxious from trauma-exposed brains.**

The cross-validated AUC statistic derived from our proposed CNN (0.80±0.02) was better than the other four tested classifiers: support vector machine (SVM) with linear kernel (0.66±0.03), SVM with RBF kernel (0.65±0.03), Gaussian process (GP) classifier with RBF kernel (0.64±0.03), and random forest (RF, 0.69±0.03). Box plots show the AUC statistics derived from 100 random selections in 5-fold cross-validation.


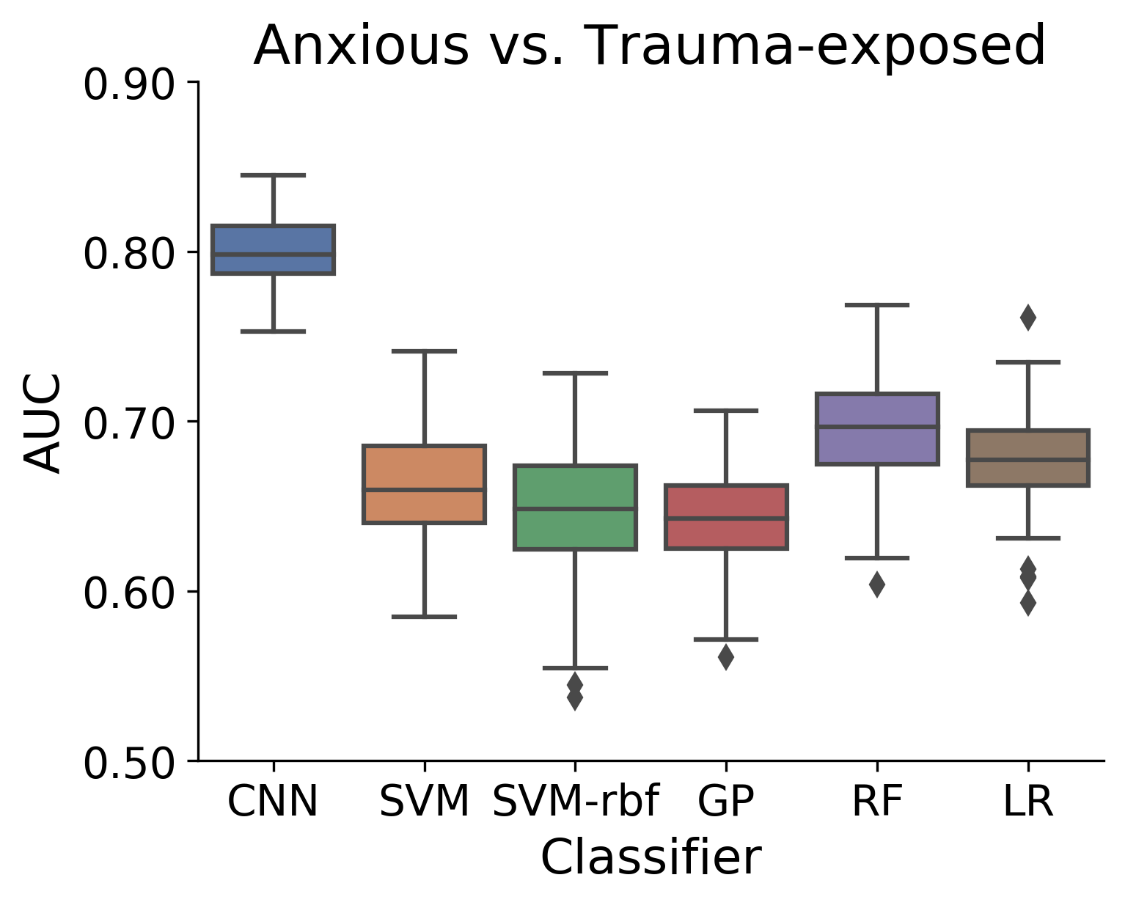


**Figure S3.** **Specificity analysis of the fear network.**

**A.** Distributions of correlation between prediction score and ASI for controls based on brain activations within the 10-node fear network, or 10 regions within the somotomotor network, or 10 randomly selected brain regions for Controls vs. Anxious and **B.** Controls vs. Trauma-exposed. **C.** Mean correlation values were shown when a specific number of regions within the fear network were replaced by a matched number of randomly selected brain regions for Controls vs. Anxious and **D.** Controls vs. Trauma-exposed.


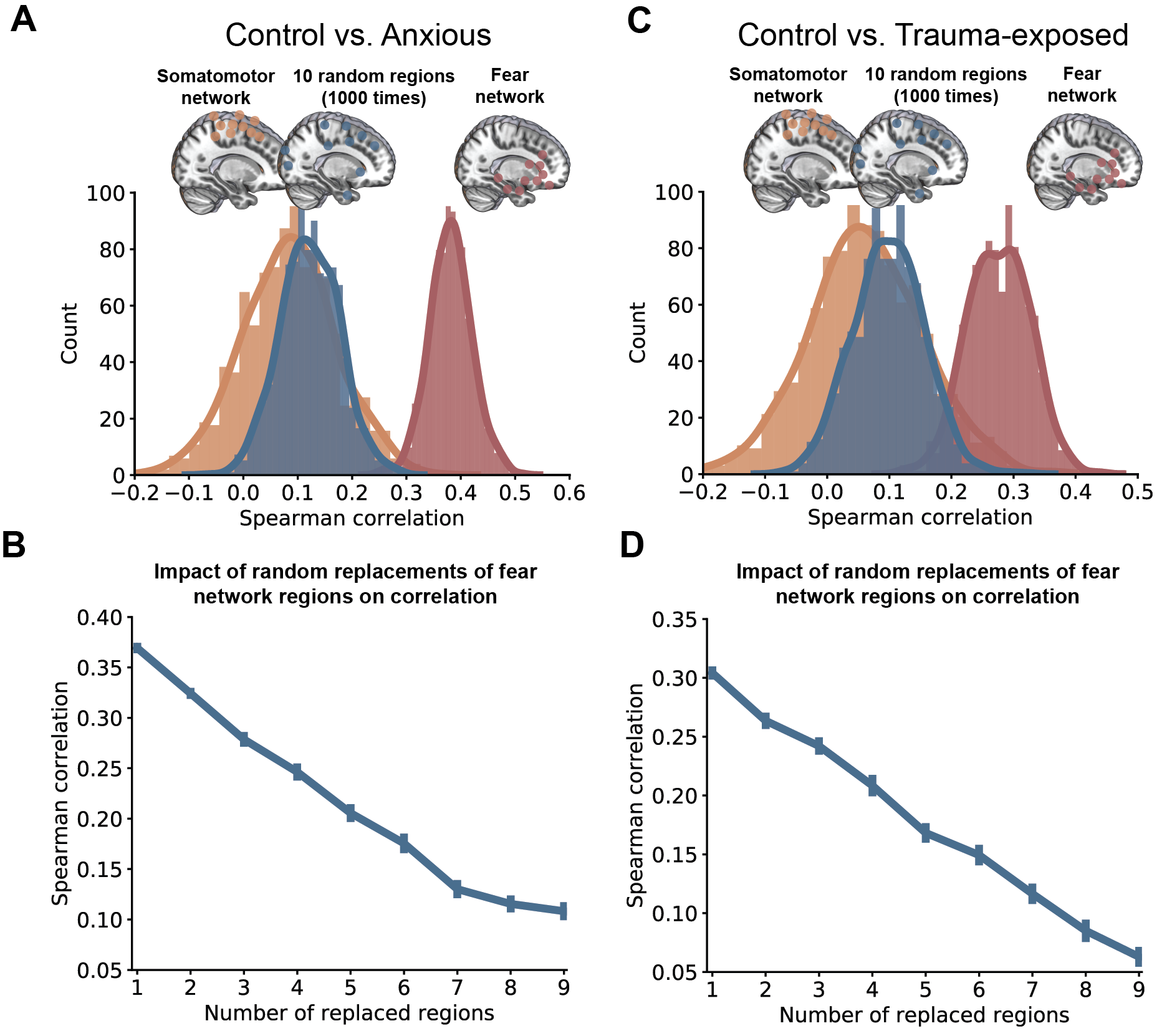


**Figure S4.** **AUCs derived from different classifiers with additional feature selection procedure. A.** Control vs. Anxious classification. **B.** Control vs. Trauma-exposed classification.


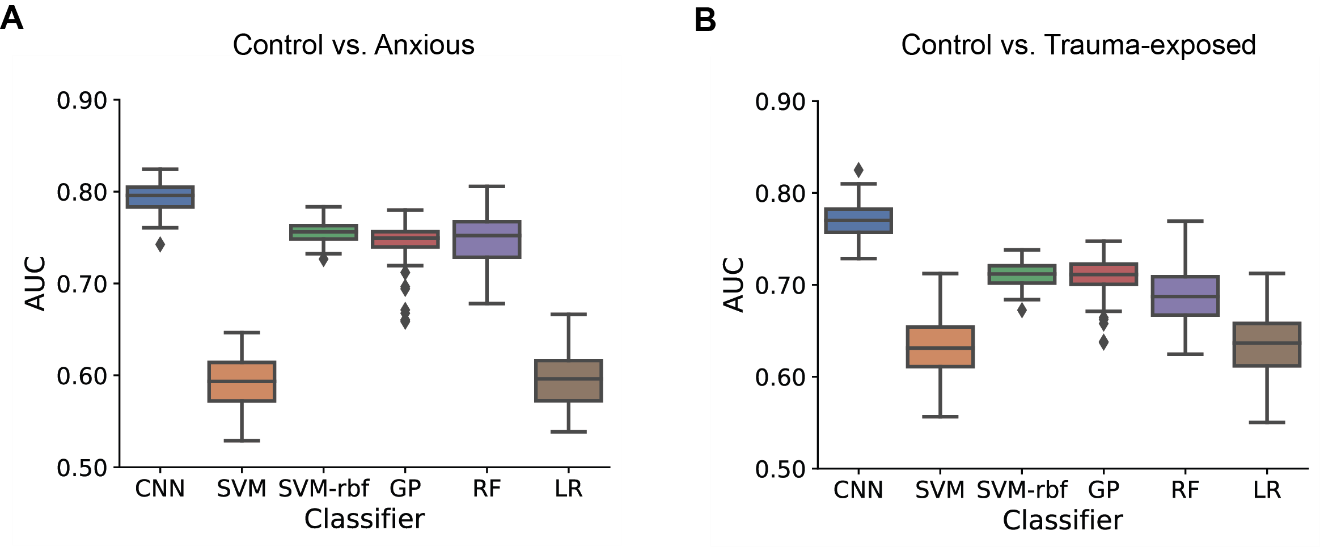


**Figure S5.** **Percentage of specific region being selected across 100 repetitions of cross-validation.** **A.** Control vs. Anxious classification. **B.** Control vs. Trauma-exposed classification. Candidates of regions including those from the fear network, and angular gyrus (ANG), cerebellum (CBM), inferior middle frontal gyrus (IMF), middle frontal gyrus (MFG), postcentral gyrus (postCG), precentral gyrus (preCG), precuneus (PCU), superior middle frontal gyrus (SMF), supplementary motor area (SMA), supramarginal gyrus (SMG).


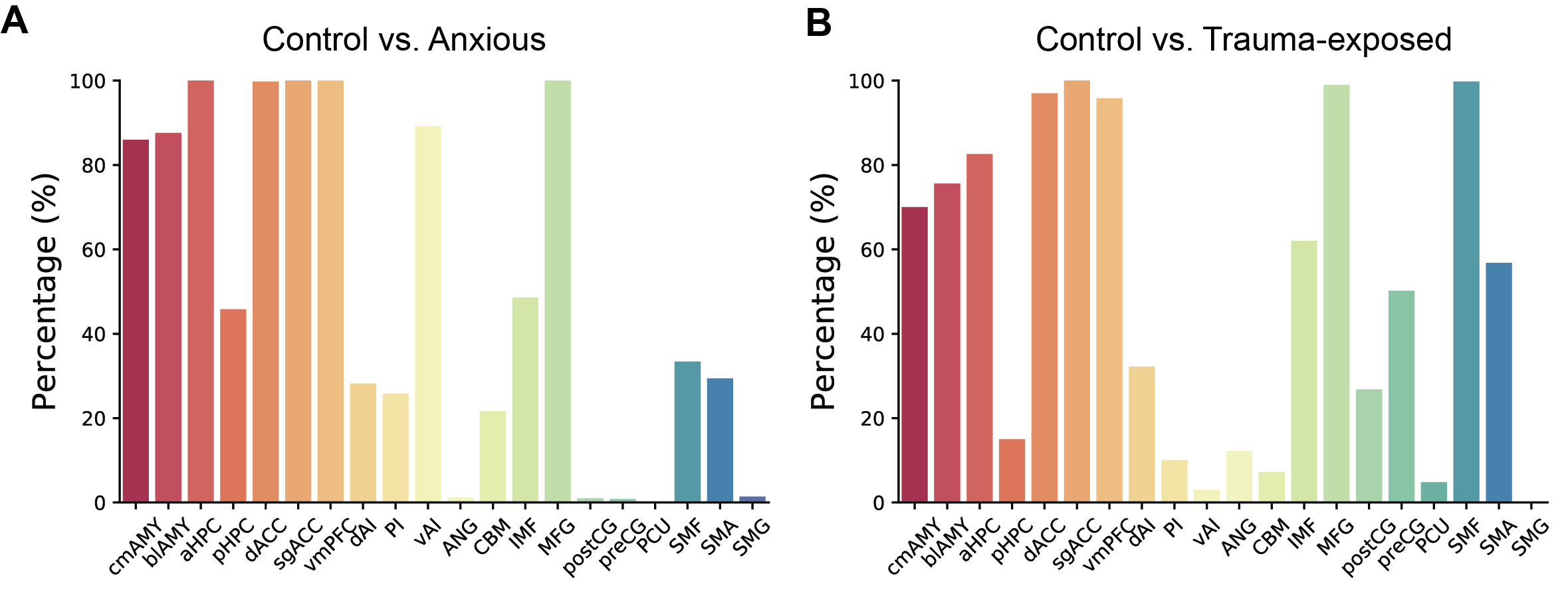


**Figure S6. AUCs derived from different kind of brain regions. A.** Control vs. Anxious classification. **B.** Control vs. Trauma-exposed classification. Fear: the fear network; Visual: 10 regions within the visual network; Others: 10 regions derived from the meta-analysis of fear conditioning.


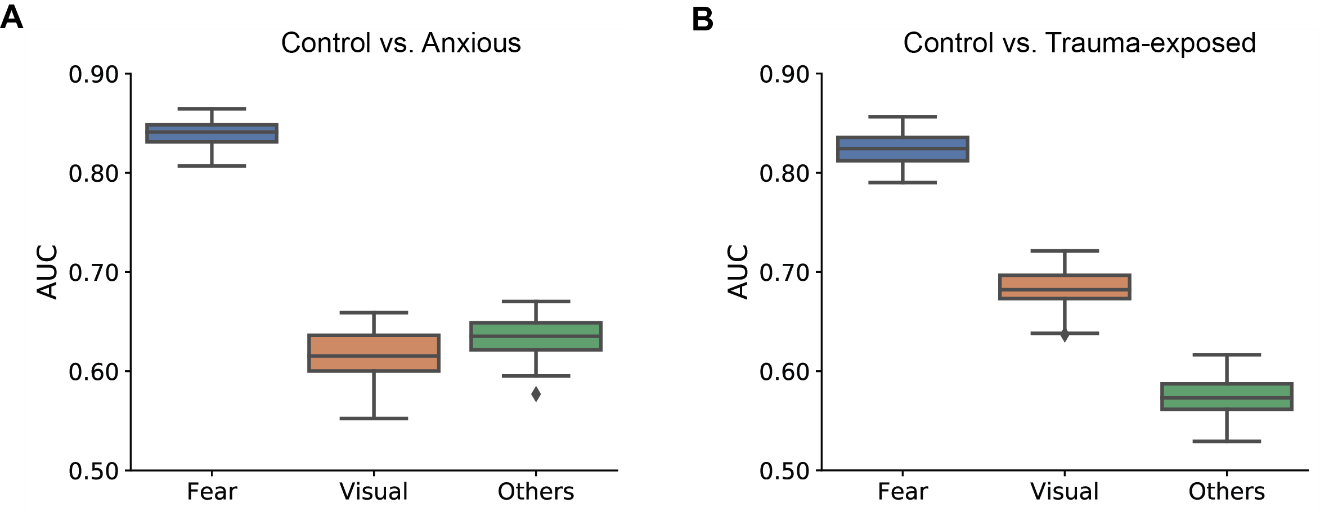


**References**

1. Milad MR, Pitman RK, Ellis CB, et al. Neurobiological Basis of Failure to Recall Extinction Memory in Posttraumatic Stress Disorder. *Biological Psychiatry*. 2009;66(12):1075-1082. doi:10.1016/j.biopsych.2009.06.026

2. Norrholm SD, Jovanovic T, Olin IW, et al. Fear Extinction in Traumatized Civilians with Posttraumatic Stress Disorder: Relation to Symptom Severity. *Biol Psychiatry*. 2011;69(6):556-563. doi:10.1016/j.biopsych.2010.09.013

3. Marin M-F, Zsido RG, Song H, et al. Skin Conductance Responses and Neural Activations During Fear Conditioning and Extinction Recall Across Anxiety Disorders. *JAMA Psychiatry*. 2017;74(6):622. doi:10.1001/jamapsychiatry.2017.0329

4. Marin M-F, Hammoud MZ, Klumpp H, Simon NM, Milad MR. Multimodal Categorical and Dimensional Approaches to Understanding Threat Conditioning and Its Extinction in Individuals With Anxiety Disorders. *JAMA Psychiatry*. Published online February 5, 2020. doi:10.1001/jamapsychiatry.2019.4833

5. Marin M-F, Song H, VanElzakker MB, et al. Association of Resting Metabolism in the Fear Neural Network With Extinction Recall Activations and Clinical Measures in Trauma-Exposed Individuals. *Am J Psychiatry*. 2016;173(9):930-938. doi:10.1176/appi.ajp.2015.14111460

6. Milad MR, Orr SP, Lasko NB, Chang Y, Rauch SL, Pitman RK. Presence and acquired origin of reduced recall for fear extinction in PTSD: Results of a twin study. *J Psychiatr Res*. 2008;42(7):515-520. doi:10.1016/j.jpsychires.2008.01.017

7. Milad MR, Wright CI, Orr SP, Pitman RK, Quirk GJ, Rauch SL. Recall of Fear Extinction in Humans Activates the Ventromedial Prefrontal Cortex and Hippocampus in Concert. *Biol Psychiatry*. 2007;62(5):446-454. doi:10.1016/j.biopsych.2006.10.011

8. Milad MR, Quirk GJ, Pitman RK, Orr SP, Fischl B, Rauch SL. A Role for the Human Dorsal Anterior Cingulate Cortex in Fear Expression. *Biol Psychiatry*. 2007;62(10):1191-1194. doi:10.1016/j.biopsych.2007.04.032

9. Garfinkel SN, Abelson JL, King AP, et al. Impaired Contextual Modulation of Memories in PTSD: An fMRI and Psychophysiological Study of Extinction Retention and Fear Renewal. *J Neurosci*. 2014;34(40):13435-13443. doi:10.1523/JNEUROSCI.4287-13.2014

10. Roy AK, Shehzad Z, Margulies DS, et al. Functional connectivity of the human amygdala using resting state fMRI. *NeuroImage*. 2009;45(2):614-626. doi:10.1016/j.neuroimage.2008.11.030

11. Tzourio-Mazoyer N, Landeau B, Papathanassiou D, et al. Automated Anatomical Labeling of Activations in SPM Using a Macroscopic Anatomical Parcellation of the MNI MRI Single-Subject Brain. *NeuroImage*. 2002;15(1):273-289. doi:10.1006/nimg.2001.0978

12. Deen B, Pitskel NB, Pelphrey KA. Three Systems of Insular Functional Connectivity Identified with Cluster Analysis. *Cereb Cortex*. 2011;21(7):1498-1506. doi:10.1093/cercor/bhq186

13. Jianping Z, Inderjeet M. kNN approach to unbalanced data distributions: a case study involving information extraction. In: *Proceedings of Workshop on Learning from Imbalanced Datasets*. Vol 126. ; 2003.

14. Pedregosa F, Varoquaux G, Gramfort A, et al. Scikit-learn: Machine Learning in Python. *J Mach Learn Res*. 2011;12(Oct):2825-2830.

15. Schaefer A, Kong R, Gordon EM, et al. Local-Global Parcellation of the Human Cerebral Cortex from Intrinsic Functional Connectivity MRI. *Cereb Cortex*. 2018;28(9):3095-3114. doi:10.1093/cercor/bhx179

16. Fullana MA, Harrison BJ, Soriano-Mas C, et al. Neural signatures of human fear conditioning: an updated and extended meta-analysis of fMRI studies. *Mol Psychiatry*. 2016;21(4):500-508. doi:10.1038/mp.2015.88
